# Experimental study of tendon sheath repair via decellularized amnion to prevent tendon adhesion

**DOI:** 10.1371/journal.pone.0205811

**Published:** 2018-10-16

**Authors:** Chunjie Liu, Kunlun Yu, Jiangbo Bai, Dehu Tian, Guoli Liu

**Affiliations:** 1 Department of Orthopedics, Tangshan Workers Hospital, Tangshan City, Hebei Province, P. R. China; 2 Department of Hand Surgery, The Third Affiliated Hospital Of Hebei Medical University, Shijiazhuang, Hebei Province, P. R. China; 3 Department of Orthopedics, The Second Hospital Of Tangshan, Tangshan City Hebei Province, P. R. China; Mayo Clinic Minnesota, UNITED STATES

## Abstract

The adhesion of tendon and surrounding tissue is the most common complication after repairing an injured tendon. The injured flexor tendons in zone II are frequently accompanied by tendon sheath defects, which lead to poor recovery. A variety of biological and non-biological materials have been recently used for repair or as substitute for tendon sheaths to prevent tendon adhesion. However, non-biological materials, such as polyethylene films, have been used to prevent tendon adhesions by mechanical isolation. The possibility of tendon necrosis and permanent foreign body remains due to the lack of permeability and the obstruction of nutrient infiltration. The natural macromolecule amniotic membrane derived from organisms is a semi-permeable membrane with the following characteristics: smooth; without vascular, nerve, and lymphatic; and rich in matrix, cytokines, enzymes, and other active ingredients. The unique structure of this membrane makes it an ideal biomaterial. In the experiment in Henry chicken, the model of tendon sheath defect and the flexor digitorum tendon in zone II was established and randomly divided into control group, medical membrane group, and decellularized amniotic membrane group. Samples were obtained at the 2nd, 4th, 8th, and 12th week after operation. General, histological, and biomechanical tests were performed to investigate the preventive effect of repaired tendon sheath by decallularized amniotic membrane. Experimental results showed the following: the amniotic membrane group and the medical membrane group had mild inflammatory reaction and tissue edema, and nearly no adhesion was observed in the surrounding tissue; the fibroblast-like cells were distributed in layers under the light microscope; the amniotic membrane group was denser than the medical membrane group cells, and numerous fibroblasts were disorganized in the control group. Biomechanical measurements showed that the sliding distance of tendon, the total flexion angle of the toes, and the tendon maximum tensile breaking strength at the early postoperative were significantly better than in the control group. Through this experiment, the amniotic membrane, as a natural biological substitute material in the construction of tendon sheath, can effectively inhibit exogenous healing and promote endogenous healing to prevent tendon adhesion.

## Introduction

The adhesion of tendon and surrounding tissue is the most common complication after repairing an injured tendon. The injured flexor tendons in zone II are frequently accompanied by tendon sheath defects, which lead to poor recovery[1]. With the theory of tendon endogenous healing and the comprehensive understanding of tendon sheath on tendon nutrition and protection, a variety of biological and non-biological materials have been recently used for repair or as substitute for tendon sheath to prevent tendon adhesion. However, due to different physical and chemical properties of these materials, their mechanisms of action and clinical effects are different. Biological materials include the tissue around the tendon, vein, and fascia transplantation[2–4]. Adopting these materials requires sacrificing other body tissues and cause new wounds; non-biological materials include polyethylene membranes, silicone membranes, cellophane, and absorbable gelatin sponges, which are obtained through mechanical isolation to achieve the purpose. With the in-depth tendon repair research, although such non-biological material membranes can isolate tissue from adhesion, these membranes have increased tendon necrosis and the possibility of permanent foreign body residue due to non-permeability, thereby impeding the penetration of nutrients[5].

The natural macromolecule amniotic membrane derived from organisms is a semi-permeable membrane characterized by the following: smooth; without vascular, nerve, and lymphatic; and rich in matrix, cytokines, enzymes, and other active ingredients. The unique structure of this membrane makes it an ideal biomaterial[6, 7]. However, the fresh amnion is unsuitable for storage and transportation and has a certain degree of immunogenicity, thus limiting its use in clinical practice[8, 9]. Many local and foreign scholars have recently used fresh amniotic membrane in clinical practice. Weak rejection, such as local cysts, has also been reported. Therefore, removal of the cellular constituents of the amniotic membrane can minimize its immune-inducing activity. In this experiment, the epithelial cell layer, fibroblast layer, and spongy layer of the enzyme-treated decellularized amniotic membrane were all removed, thereby leaving only the basal and dense layers. The texture was soft, and the biocompatibility was good. No evident immune induction activity was observed. In the experiment in Henry chicken, the model of tendon sheath defect and the flexor digitorum tendon in zone II was established to investigate the preventive adhesion effect of repaired tendon sheath by decellularized amniotic membrane.

## Materials and methods

### Designing randomized control animal experiments

#### Time and place

The experiment was completed at the Animal Experimental Center of the Third Hospital of Hebei Medical University from May to July 2017.

#### Materials

Absorbable medical membrane (poly-DL-lactic acid PDLLA, Chengdu Dikang Zhongke Biomedical Material Co., Ltd.); ElectroForce 3520 biomechanical testing (BOSE, USA).

Experimental animals: A total of 60 male 6-month-old hens with body weight of (2.0 ± 0.05) kg were provided by the Animal Center of Hebei Medical University. A total of 60 hen chickens were individually numbered and randomly divided into the following three groups: decellularized amniotic membrane group, absorbable membrane group, and control group. Each group comprised 20 chickens.

Decellularized amniotic membrane was provided by the Department of Obstetrics and Gynecology from the Third Hospital of Hebei Medical University. The HBV, HCV, HIV, syphilis, and gonorrhea were all negative after maternal consent and the serological examination of the parturient. The fresh amniotic membrane was washed three times with sterile phosphate-buffered saline containing 50 μg/mL of penicillin and 50 μg/mL of streptomycin, deprived of the spongy layer, and cut into 1.0 × 0.5 cm pieces. The epithelial cells were removed by incubation in ethylenediaminetetraacetic acid 0.05% (Invitrogen, Germany) at 37°C for 2 h and then gently scraped with a cell scraper under a microscope. The complete removal of epithelial cells was confirmed using hematoxylin and eosin (H&E) (Sigma Aldrich, Germany) staining[10]. The prepared acellular amniotic membrane was cut into 1.0cm*0.5cm in a sterile clean room, placed in a sterile aluminum foil film packaging bag, vacuum sealed, and sterilized with ethylene oxide for 6 hours.

The study was approved by the Ethics Board of the Third Hospital of Hebei Medical University and was conducted in accordance with the institutional guidelines for the care and treatment of animals.

#### Experimental methods

**Fig 1:**
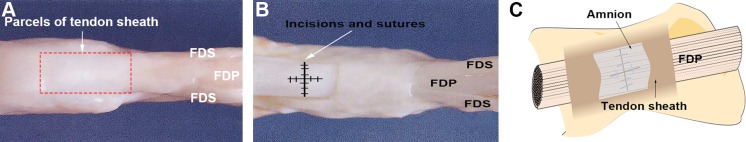
Similarity of chicken toe tendons to human digital flexor tendons. Flexor digitorum superficialis(FDS), flexor digitorum profundus (FDP). A and B quoted and modified from Xu Y, Tang JB. J Hand Surg. 2003;28A:944–1001[11]. A. The red box is the range of tendon sheath around the tendon. B. The incision and suture of the tendon after stripping part of the tendon sheath. C. Schematic diagram of acellular amniotic membrane repairing tendon sheath.

The third toe of Leheng chicken was used to prepare an injured tendon and a tendon sheath defect model. After being anesthetized by intramuscular injection of 25 mg/kg ketamine, the animal was subjected to a longitudinal incision of approximately 2.0 cm proximal to the lateral interphalangeal joint of the third toe, and the subcutaneous tissue was dissected to expose the tendon sheath and flexor digitorum tendon. Approximately 1.0 cm × 0.5 cm part of the toe tendon sheath and the superficial flexor tendon was resected, and the deep flexor tendon was freed. A 2/3 circumference was cut across the ramp, and 0.5 cm was cut from the transverse to the proximal/distal longitudinal direction. The severed tendon was then sutured with 5–0 noninvasive line "8". In the amniotic membrane group and medical membrane group, the appropriate size of the decellularized amniotic membrane and the medical film were laid on the defect of the tendon sheath, and the edges were sutured and fixed(Fig 1). The tendon sheath defect in the control group was not repaired. No surgery was performed on the healthy side foot. The postoperative surgical incision comprised aseptic dressings, and no fixation was performed. After the experiment, the animals were housed in cages and fed with oxytetracycline for three days after the operation, and the sutures were removed two weeks after the operation.

### Key Indicators

Evaluation of tendon adhesion: The basic condition of the experimental animals was observed regularly after surgery. Fifteen experimental animals were sacrificed at 2, 4, 8, and 12 weeks after surgery. The third toe was obtained, the skin and subcutaneous tissue were set off, and the degree of adhesion between the tendon and the surrounding tissues and the healing degree of tendon were observed. Tendon adhesion (macroscopic and microscopic) was evaluated due to the criteria defined by Tang. [12] (Tables 1 and 2).

**Table 1:** Criteria described by Tang for macroscopic evaluation of adhesions.

	Points	Adhesion appearance
Length	0	No adhesion
	1	Localized, <10mm longitudinal
	2	10–15mm
	3	Intense, >15mm
Characteristics	0	No adhesion
	1	Loose, elastic, and mobile
	2	Of average thickness and mobile
	3	Thick, hard, and immobile
Grading	0	No adhesion
	1	Mild adhesion
	2	Moderate adhesion
	3	Advanced stage adhesion

**Table 2:** Criteria described by Tang for microscopic evaluation of adhesions.

Points	Features of adhesion
	Quantity
0	No apparent adhesions
1	A number of scattered filaments
2	A large number of filaments
3	Countless filaments
	Quality
0	No apparent adhesions
1	Regular, elongated, fine, and filamentous
2	Irregular, mixed, shortened, and filamentous
3	Dense, not filamentous
	Grading of adhesions
0	None
1–2	Slight
3–4	Moderate
5–6	Severe

Histological observation: After gross observation, two toes 0.5 cm × 0.5 cm in size were randomly taken from the toes and served as the center of the tendon anastomosis to reconstruct the tendon sheath. These toes were routinely fixed, embedded, sectioned, H&E stained, and histologically observed under light microscope.

Transmission electron microscopy (TEM): At the 4th and 8th weeks postoperatively, freshly formed sheaths of 1.0 mm × 1.0 mm × 1.0 mm were rapidly resected in each group of specimens, fixed with 3% glutaraldehyde, embedded in epoxy resin, sectioned, and observed under transmission electron microscope.

**Fig 2:**
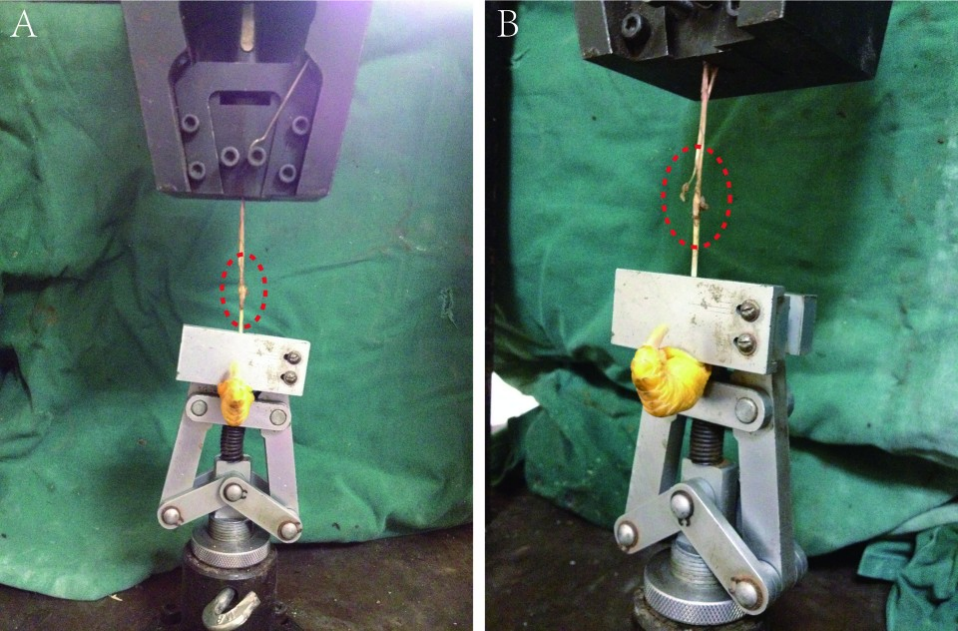
Biomechanical testing of tendons. A. Inside the red box is the tendon repaired after injury. B. The traction force gradually increased and the tendon began to break partially.

Biomechanical test: At the 2nd, 4th, 8th, and 12th weeks after surgery, the animals were sacrificed from the metacarpal and metatarsophalangeal joints. The deep flexor digitorum tendon was reserved. The tendon sliding distance, the total flexion angle, and the maximum tensile breaking strength were measured on a biomechanical experiment machine. The proximal phalanx of the toes was fixed on the experimental machine, and the reserved deep toe flexor tendon was pulled with 1N force, thereby gradually increasing the traction force to 10 N. The length of the tendon that was pulled out of the tendon sheath was marked and measured. The flexion angle of each interphalangeal joint was measured with a protractor, and the total flexion angle (sum of the total flexion angle of the three interphalangeal joints) was calculated thrice for each specimen to obtain the average. The tissues around the deep flexor tendon were freed, and the distal phalanx was reserved. The two ends of the deep flexor tendon were fixed on the biomechanical testing machine, and the tension was adjusted. The tension was moved at a speed of 20 mm/N until the tendon ruptured. The maximum tensile strength of the tendon was synchronously recorded(Fig 2).

### Statistical analysis

The data were statistically processed by SPSS 22.0 software. In biomechanical testing, two groups were compared using the SNK test, and P < 0.05 indicates that the difference was statistically significant. Kruskal-Wallis test and Bonfferroni test as post hoc were used in Evaluation of tendon adhesion. Bonferroni correction is used to reduce the chances of obtaining false-positive results since multiple pairwise tests are performed on a single set of data. To perform a Bonferroni correction, the alpha (𝛼) was divided by the number of comparisons being made. In our study, there were three groups and three pairwise comparisons. So we divided the alpha by three. The statistical significance level for comparison was set as 𝑝 < 0.016.

## Results

### Analysis of the number of experimental animals

The experimental animals were in good condition without surgical incision infection and inflammatory necrotic tissues. Furthermore, 60 Henchen chickens were all involved in the analysis of results.

### General observation results

2 weeks after the operation: Structures similar to tendon sheath were observed in all groups. The tendon anastomosis was expanded, and the surface remained smooth. The tissue was evidently congested and swollen. The decellularized amnion of the amniotic membrane group and the medical membrane of the medical membrane group were blurred but remained recognizable. A small amount of synovial fluid was found in the perineal space with a fibrous link in the broken end of the tendon and no adhesion to the surrounding tissue. In the control group, the surface of the tendon sheath was rough, and no synovial fluid was evident in the tendon. Numerous fibrous connective tissues were observed in the tendon anastomosis area, and removing the anastomotic mouth was easy.

4 weeks after the operation: Tissue edema in the tendon anastomosis area of each group was significantly reduced. The amniotic membrane group and the medical membrane group demonstrated complete reconstruction of the tendon sheath. No adhesion was found in the peritendon. Identifying the amniotic membrane was difficult. The tendon anastomosis was healed well, and transparent synovial fluid was observed in the peritendon; however, no adhesion or mild conglutination was found in the peritendon. In the control group, the tendons were connected by fibrous tissues with the perilineal tissues, and removing the dissection was difficult.

**Fig 3:**
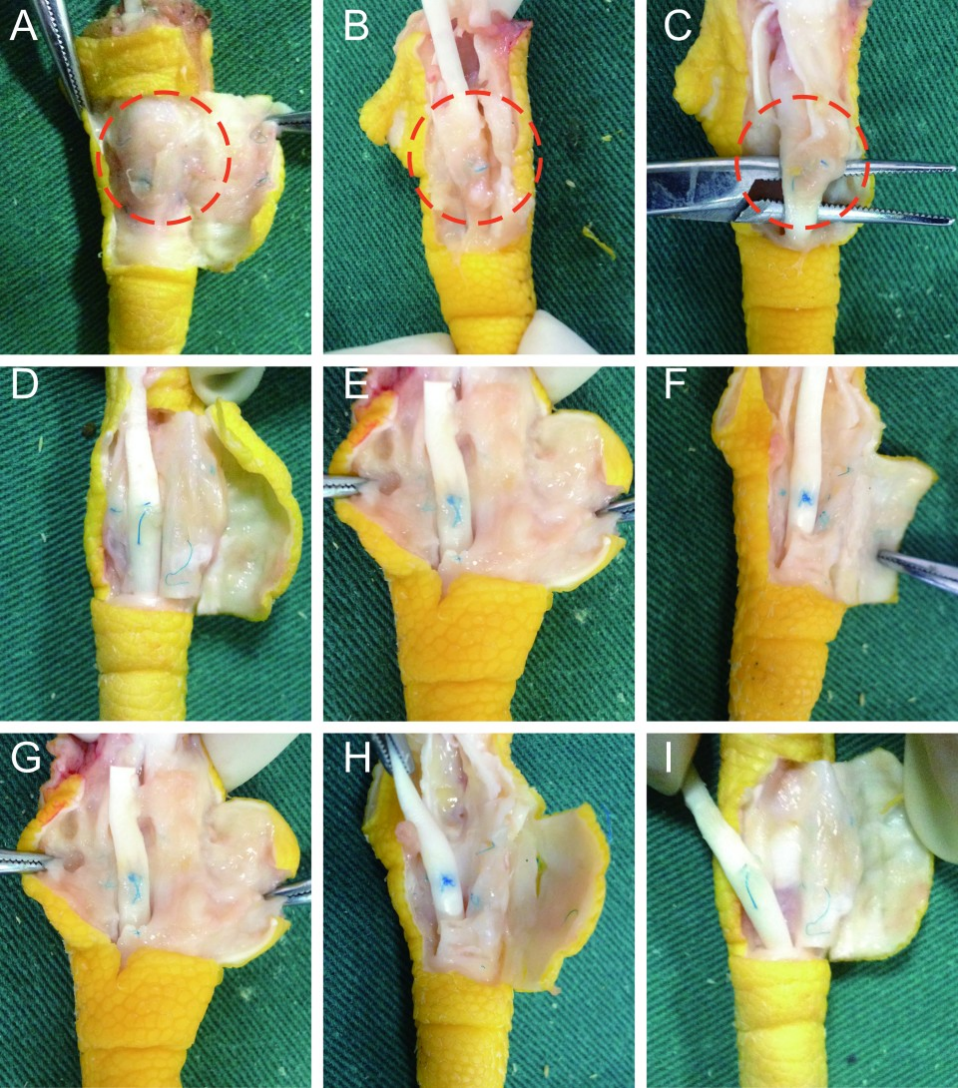
General observation of each group at the 2,4,8 week after operation. (A,B,C) No complete tendon sheath was observed in the control group. The tendon anastomosis was connected with fibrous connective tissue, which was firm and difficult to remove around the tendon tissue. The red circle refers to an adhesion formed between the tendon and the surrounding tissue. In the medical membrane group (D,E,F) and amniotic membrane group (G,H,I), the tendon sheath was reconstructed, and the tendon anastomosis healed well without adhesion. The medical membrane was vaguely visible while the amniotic membrane was unrecognizable.

8 and 12 weeks after the operation: The amniotic membrane group and the medical membrane group had intact tendon sheaths, and the tendons had healed well without adhesion, which was close to the normal structure. In the control group, the adhesion between the peritendon tissues and the tendon was severe and forming an effective slide in the tendon was difficult (Fig 3).

### Histological observations

2 weeks after operation: Bleeding, edema, and inflammatory infiltration were observed in all groups. Bleeding and inflammatory cell infiltration in the amniotic membrane group were minimal. Moderate inflammatory cell infiltration and edema were evident in the medical membrane group. In the control group, numerous inflammatory cells were infiltrated with bleeding and edema.

4 weeks after surgery: The number of inflammatory cells in each group was significantly reduced. The amniotic membrane group and medical membrane group showed a layered distribution of fibroblast-like cells. The amniotic membrane group was denser than the medical membrane group cells. Numerous disorganized fibroblasts were observed in the control group.

**Fig 4:**
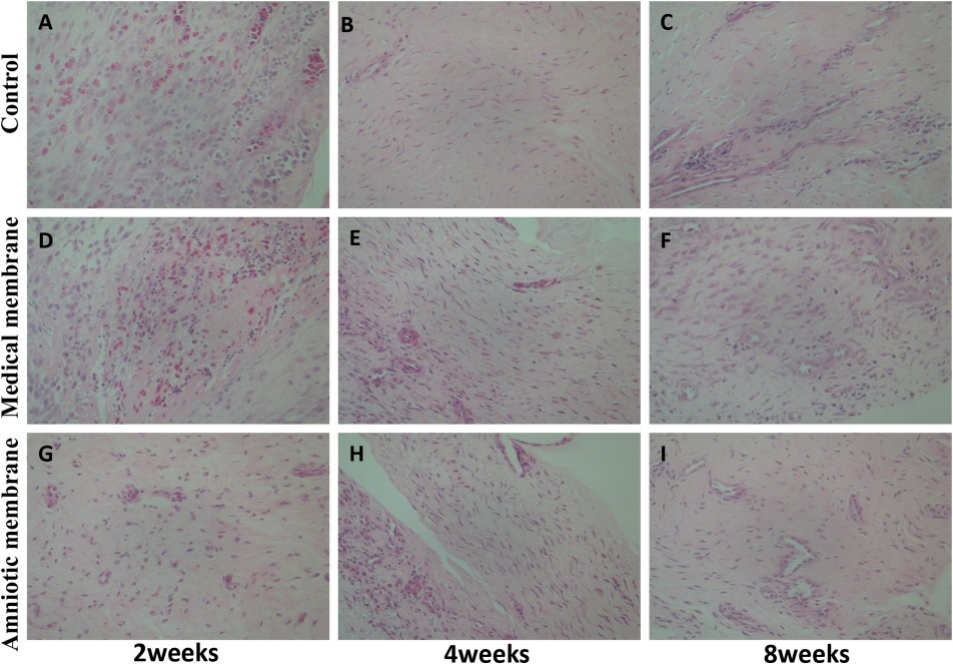
Histological observation of the tendon sheath defection in each group at the 2nd, 4th, and 8th week after operation (hematoxylin–eosin staining, x 400). At the 2nd week, each group was filled with hyperemia, edema, and inflammatory cell infiltration, but the amniotic membrane group (G) was the lightest. At the 4th week, inflammatory cells were significantly reduced than before. In the medical membrane group (E) and the amniotic membrane group (H), fibroblast cells were layered, and the amniotic membrane group was dense. In the control group (B), numerous fibroblasts were disorderly distributed. At the 8th week, the fibroblasts in the medical membrane group (F) and the amniotic membrane group (I) were arranged neatly, and the structure was dense. In the control group (C), the structure of tendon sheath was loose, and the distribution of fibroblasts was disorganized.

8 and 12 weeks after operation: The amniotic membrane and the medical membrane were arranged in a layered manner and the structure was dense. The control group comprised disordered distribution of fibrocytes(Fig 4).

### TEM observations

4 weeks after operation: The tendon cells proliferated, the nucleus was large and round, and the chromatin was increased. Double nuclei and many organelles were found. Numerous synovial cells were found in the amniotic group, and the type B cells were more evident than the type A cells. The dilatation of type B cells was also evident. Many dilated rough endoplasmic reticulum, mitochondria, and lysosomes were observed in the matrix, and the endoplasmic reticulum was filled with secretions. The results of electron microscopy in the medical membrane group were similar to those in the amniotic membrane, but the number of synovial cells was lower than that in the amniotic membrane group. The content of synovial cells in the control group was significantly lower than that in the first two groups, and the content of the rough endoplasmic reticulum in the matrix of B synovial cells was low and the extension degree was not evident.

**Fig 5:**
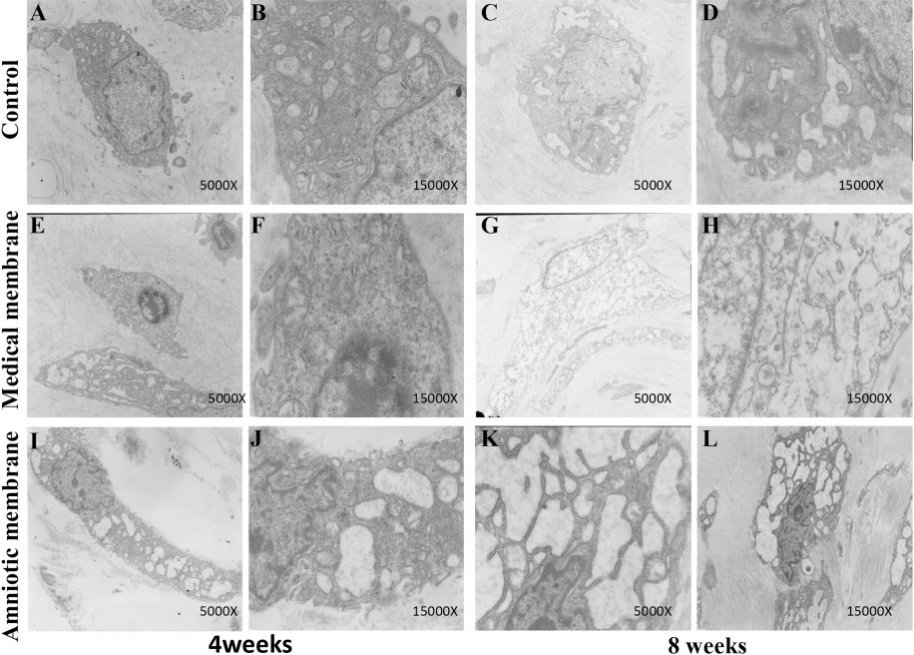
Transmission electron microscopy observations of the defection area of tendon sheath at the 4th and 8th week after operation. The number of synovial cells in the control group (A–D) was significantly lower than that of the two other groups. Moreover, the content of the rough endoplasmic reticulum in the cell matrix was low, and the extension degree was not evident. The medical membrane group (E–H) was similar to the amnion group, but the number of synovial cells was lower than that of the amnion group. Numerous synovial cells were observed in the amniotic group (I–L). Furthermore, numerous dilated rough endoplasmic reticulum and secretions in the endoplasmic reticulum were found in the matrix.

8 weeks after operation: The tendon cells were slender, the nucleus was similar to a long rod, the organelles evidently decreased compared with the prophase, and the microstructure of the collagen was clear. In the amniotic membrane and medical membrane groups, numerous B synovial cells were observed and A synovial cells were rare. The extent of endoplasmic reticulum expansion in the group is poor compared with that of the two previous groups (Fig 5).

### Evaluation of tendon adhesion

There was more or less adhesion in all groups. There are statistically significant differences between amniotic membrane group and control group in the macroscopic evaluation of adhesion, medical membrane group and control group, amniotic membrane group and control group in the microscopic evaluation of adhesion at the 12th week after operation (𝑝 < 0.016). The detailed results are shown in Tables 3–6.

**Table 3:** Rates of macroscopic and microscopic adhesion of groups according to Tang criteria after 2 weeks after operation.

Adhesion (%)								
	Macroscopic adhesion (n = 15)				Microscopic adhesion (n = 15)			
	None	Slight	Moderate	Severe	None	Slight	Moderate	Severe
Control group	0	40	40	20	0	40	40	20
Medical membrane group	0	80	20	0	0	80	20	0
Amniotic membrane group	0	80	20	0	0	80	20	0

**Table 4:** Rates of macroscopic and microscopic adhesion of groups according to Tang criteria after 4 weeks after operation.

Adhesion (%)								
	Macroscopic adhesion (n = 15)				Microscopic adhesion (n = 15)			
	None	Slight	Moderate	Severe	None	Slight	Moderate	Severe
Control group	0	20	60	20	0	40	60	0
Medical membrane group	0	40	60	0	0	80	20	0
Amniotic membrane group	0	80	20	0	0	100	0	0

**Table 5:** Rates of macroscopic and microscopic adhesion of groups according to Tang criteria after 8 weeks after operation.

Adhesion (%)								
	Macroscopic adhesion (n = 15)				Microscopic adhesion (n = 15)			
	None	Slight	Moderate	Severe	None	Slight	Moderate	Severe
Control group	0	0	60	40	0	20	60	20
Medical membrane group	0	40	60	0	0	80	20	0
Amniotic membrane group	0	80	20	0	0	80	20	0

**Table 6:** Rates of macroscopic and microscopic adhesion of groups according to Tang criteria after 12 weeks after operation.

Adhesion (%)								
	Macroscopic adhesion (n = 15)				Microscopic adhesion (n = 15)			
	None	Slight	Moderate	Severe	None	Slight	Moderate	Severe
Control group	0	0	40	60	0	0	20	80
Medical membrane group	0	40	60	0	0	80	20	0
Amniotic membrane group	0	80	20	0	0	100	0	0

### Biomechanical test results

Sliding distance of tendon: No significant difference was observed in the sliding distance between the amniotic membrane group and the medical membrane group at each time point after the operation, which was higher than in the control group (P < 0.05).

Total flexion angle of the toes: There was no significant difference in the total flexion angle of the toes between the amniotic membrane group and the medical membrane group at each time point after the operation, which was higher than that of the control group (P < 0.05).

**Fig 6:**
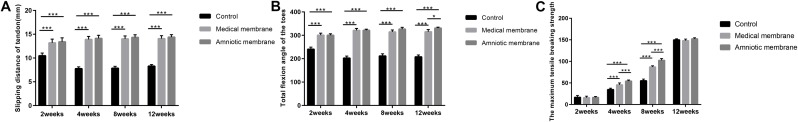
Comparison of biomechanical results at different time points after operation. The SNK method was used to compare the two groups, and the P < 0.05 indicated that the difference was statistically significant. (A) No significant difference was found in the slipping distance between the amniotic membrane group and the medical membrane group at each time point, which was higher than that of the control group (P < 0.05). (B) No significant difference was found in the angle of total flexion between the amniotic membrane group and the medical membrane group at each time point after the operation, which was higher than that of the control group (P < 0.05). (C) No significant difference was found in the maximum tensile breaking strength between the 2nd and 12th week after operation (P > 0.05). At the 4th and 8th week, the maximum tensile breaking strength of the amniotic membrane group and medical membrane group was higher than that of the control group (P < 0.05), whereas the amniotic membrane group was higher than that of the medical membrane group (P < 0.05).

Tendon maximum tensile breaking strength: At the 2nd and 12th weeks after surgery, no significant difference was observed in the maximum tensile rupture strength of the tendon (P > 0.05). At the 4th and 8th week after operation, the maximum tensile breaking strength of the amniotic membrane group and the medical membrane group was higher than that of the control group (P < 0.05), and the amniotic membrane group was higher than the medical membrane group (P < 0.05) (Fig 6).

## Discussion

In the field of hand surgery, 30% of cases suffer from tendon injury alone or associated with tendon injury. The anatomical structure of the flexor tendon in the hand zone II is complex and delicate, mostly presenting tendon sheath defects. Thus, tendon adhesion is prone to occur after tendon repair[13]. Exogenous and endogenous methods are available for tendon healing. In exogenous healing of tendons, fibroblasts grow from the surrounding tissue to the ends of the tendons, thereby forming the adhesion of the tendons and surrounding tissues, hindering the normal sliding of tendons, and seriously affecting the motor function of the fingers. Such issues remain unsolved in the field of tendon injury repair[14, 15]. Inhibiting exogenous healing, promoting endogenous healing, reducing inflammation, controlling excessive fibroblast proliferation, and effectively recovering the tendon sliding function are keys to preventing tendon adhesion.

The role of the tendon sheath has received significant attention in the process of improving the effect of the repaired flexor digitorum tendon in zone II[16]. The current view is that the tendon sheath is not only the supporting tissue of the tendon but also has nutritious and auxiliary sliding effect on the tendon. Eikon et al. summarized the effects of tendon sheath as follows: (1) as a barrier tissue to prevent the growth of surrounding granulation tissue, thereby preventing scarring adhesions; (2) as a component of the tendon sliding mechanism; (3) the synovial fluid secreted by the synovial membrane of the tendon sheath which has a nutritive effect on the tendon[17–19]. Based on the preceding understanding, restoring tendon sheath integrity not only protects the tendon nutrition but also promotes the formation of new tendon tissues and blood vessels and accelerates tendon healing. Preventing the infiltration of granulation tissue and providing a good sliding device for the tendon as a barrier are beneficial to improve the tendon sliding function after tendon repair. However, suturing the tendon sheath in situ after injury is difficult because large tendon sheath defects are frequently present. Determining the kind of tissue or material for repairing the missing tunica vaginalis is one of the difficulties of tendon repair. Many biological and nonbiological materials are presently used as tendon sheath substitute to reconstruct the damaged tendon sheath.

Non-biological material films frequently used clinically, such as polylactic acid, mainly achieve their purpose through mechanical isolation. With further research of tendon repair, such abiotic material membrane can isolate tissues and prevent adhesion but can also increase the possibility of tendon necrosis and permanent foreign body residues due to non-permeability, thereby impeding the penetration of nutrients[20]. According to the previous treatment methods, the preceding cases mentioned are likely to be broken again, be poorly healed, be stiff, and demonstrate severe adhesion of the tendons and surrounding tissues, thereby resulting in limited finger function. The ideal material for preventing tendon adhesion should meet the following requirements: 1) can be absorbed without leaving any foreign body in vivo; 2) can be easily obtained and has good histocompatibility; 3) has good permeability and does not affect the quality of tendon healing while blocking the exogenous healing of tendon; and 4) contains factors that promote cell adhesion, growth, proliferation, and differentiation.

The amniotic membrane is a semitransparent, double-layer film attached to the placental fetal surface, which evolves from the cell trophoblast. The thickness of the amniotic membrane is approximately 0.02–0.05 mm, which is the thickest basement membrane of the human body. The surface of this membrane is smooth and has no blood vessel, nerve, and lymph. The membrane surface also has certain elasticity. The decellularized amniotic membrane, which removes epithelial cell layer and only remains as the basement membrane, is a natural biological transplantation material. The main components of this membrane include a variety of collagen, glycoprotein, and proteoglycan with good histocompatibility, low antigenicity, and strong permeability and can be completely degraded in vivo. Acellular amniotic membrane has been applied in the treatment of ocular surface disease, diabetic foot, periosteal perforation, urethral tissue defect, and other skin or mucosal injuries, which has achieved satisfactory results[21, 22].

Two flexor tendons are present in the chicken toe, namely, superficial and deep flexor tendons, which have sheaths, buttons, and pulleys. The structure of the toe is similar to the human finger. Therefore, in this experiment, the deep flexor tendon of the chicken toe was used to imitate the human finger to establish the injured model of the deep flexor tendon in zone II. The structure of the hand tendon is delicate, and the function is complex. After the injury is repaired, the tendon must possess a certain tensile strength and good sliding capability to drive the joint activity. The early functional exercise is an important method to facilitate the recovery of tendon adhesions. The resection of the tendon sheath, suturing, and braking play a key role in the formation of tendon adhesions; only braking leads to moderate adhesions. Therefore, animals were not postoperatively plastered in this study to reduce adhesions. In the experiment, the tendon suture area was wrapped by the "sheaths" formed by amniotic membrane, which effectively isolated the surrounding tissues and increased the sliding of the tendon. The sheath tube is an ideal barrier to maintain the normal environment of the tendon, which is beneficial to the repaired tendon itself[23, 24].

The migration, attachment, and proliferation of tendon fibroblasts, protein synthesis, and the formation of extracellular matrix are important processes for repairing and remolding of the injured tendon. After the injury occurs, fibroblasts and inflammatory cells infiltrate from the perilineal tissue to the repair area, which strengthens the deposition of collagen fibers and serves as the material basis for tendon tissue repair; however, such action simultaneously causes adhesion between tendons and surrounding tissues. The results of this study showed that the infiltration of monocytes and neutrophils in the ruptured area was significantly less in the amniotic membrane group than that in the negative control group at the 2nd and 4th week postoperatively; the number of adhesions and the degree of adhesion were lower than those of the negative control group. The experimental results suggested that decellularized amniotic membrane has anti-inflammatory and anti-adhesive effects. The amniotic membrane by expression of interleukin-1 receptor antagonist (IL-1 Ra) reduced inflammation caused by IL-1, upregulated the release of IL-4, IL-10, matrix metalloproteinase inhibitors, and other active substances to enhance the capability to resist tissue inflammation, and promoted tissue cell repair and anti-scar effect in the injured region[25–30].

The content of collagen in the tendon, especially type I collagen, is related to the biomechanical property of repaired tendon. The tendon must be completely healed through collagen fiber deposition, molding, and maturation[31, 32]. This study proved that the order of fibroblasts and collagen fibers in the amniotic tendon group was significantly better than that in the control group at the 2nd week after operation. The collagen fiber arrangement at the 8th week was similar to the normal tendon. At the 2nd and 4th week after operation, the sliding distance of tendon, the total flexion angle of the toes, and the maximum tensile breaking strength of the amniotic membrane group were significantly higher than those of the control group. These results indicate that the mechanical strength of the repaired tendon in the early stage can meet the physiological function needs of the body.

Biomechanical test results showed that the sliding distance of tendon and the total flexion angle of the toes in the amniotic membrane group and the medical membrane group were significantly better than that in the control group; the maximum tensile breaking strength of the tendon in the amniotic membrane group was better than the two other groups at the 4th and 8th week after operation. Tendon sheath reconstruction promotes endogenous healing and optimizes the strength of tendon healing while blocking the exogenous healing of tendon. The nutrition mechanism of tendon is not only blood nutrition provided by peritendon tissue and tendon bundle vessels but also the synovial cells secreting synovial fluid, which is an important nutritional pathway for intrathecal tendons. Under the transmission electron microscope, the rough endoplasmic reticulum and Golgi apparatus in the matrix of type B synovial cells in the decellularized amniotic membrane group were highly dilated, the medical membrane group was slightly weaker, and the control group was the least evident. By contrast, the type B synovial cells were the main cells which secreted synovial fluid[33, 34].

The amniotic membrane is a semi-permeable membrane characterized by the following: smooth; without vascular, nerve, and lymphatic; rich in matrix, cytokines, enzymes, and other active ingredients. The unique structure of this membrane makes it an ideal biomaterial. The study showed that the amniotic membrane was used to repair the tendon sheath and isolate the surrounding tissue to prevent or reduce adhesion between the tendon and the surrounding tissue. The damaged tissues are shaped in the direction of the scaffold, and the arrangement and remolding of the collagen fibers in the tendon cells are improved[35]. The nutrients penetrate through a semipermeable membrane, promote repair of damaged cell, and effectively promote tendon endogenous healing. Basic fibroblast growth factor, hepatocyte growth factor, and transforming growth factor-beta (TGF-β) promote stem cell differentiation, transformation, proliferation, and migration. It inhibits the expression of TGF-β receptor and the proliferation and differentiation of fibroblast; it also downregulates the expression of β-smooth muscle actin, fibronectin, and integrin, decreases the biological function of fibroblasts, and reduces fibrosis and scar formation[36–38].

The experiments showed that the amniotic membrane was thin, and the mechanical strength was low, thereby making it easy to tear. In the experiment, a single-layer amniotic membrane was used to reconstruct the tendon sheath. The integrity of some amniotic membrane was frequently damaged during the process of tendon sliding. The acellular amniotic membrane removes its own cellular components, which can be combined with a variety of cells for culture, thereby making the acellular amniotic membrane an ideal scaffold for tissue engineering[21, 39].

Amniotic membrane, as a natural biological substitute, has unique advantages in constructing tendon sheath, inhibiting external healing, promoting endogenous healing, and preventing tendon adhesion. With the further research on the biological characteristics of amniotic membrane and the further development in medical, biological, and tissue engineering applications, the immunity, anti-inflammatory, antibacterial, and anti-adhesion of amniotic membrane will be comprehensively reflected in clinical application.

## Supporting information

S1 Table The sliding distance of the tendon. The original data of the sliding distance of the tendon were measured at 2, 4, 8, and 12 weeks after surgery.
(DOCX)

S2 Table The total flexion angle of the toes. The original data of the total flexion angle of the toes were measured at 2, 4, 8, and 12 weeks after surgery.
(DOCX)

S3 Table The tendon maximum tensile breaking strength. The original data of The tendon maximum tensile breaking strength were measured at 2, 4, 8, and 12 weeks after surgery.
(DOCX)
