# Supplementary material for: Experimental study of tendon sheath repair via decellularized amnion to prevent tendon adhesion
Source: PLoS One. 2018 Oct 16;13(10):e0205811. doi: 10.1371/journal.pone.0205811 (PMC6191119; doi:10.1371/journal.pone.0205811)
Supplement: S1 Table — The original data of the sliding distance of the tendon were measured at 2, 4, 8, and 12 weeks after surgery. (DOCX) [file pone.0205811.s001.docx]

| The sliding distance of tendon（mm） | | | | |
| --- | --- | --- | --- | --- |
| amniotic membrane group | 2weeks | 4weeks | 8weeks | 12weeks |
|  | 12.67 | 13.69 | 14.07 | 14.11 |
|  | 13.05 | 13.99 | 14.18 | 14.22 |
|  | 13.2 | 13.66 | 14.02 | 14.05 |
|  | 13.27 | 14.08 | 14.15 | 14.22 |
|  | 14.85 | 15.23 | 15.31 | 15.32 |

S1 Table: The sliding distance of the tendon. The original data of the sliding distance of the tendon were measured at 2, 4, 8, and 12 weeks after surgery.

|  | | | | |
| --- | --- | --- | --- | --- |
| medical membrane group | 2weeks | 4weeks | 8weeks | 12weeks |
|  | 12.45 | 13.45 | 13.55 | 13.57 |
|  | 12.81 | 13.81 | 13.92 | 14.02 |
|  | 13.01 | 13.45 | 13.61 | 13.65 |
|  | 13.21 | 13.83 | 14.03 | 14.05 |
|  | 14.5 | 14.99 | 15.05 | 15.1 |

|  | | | | |
| --- | --- | --- | --- | --- |
| control group | 2weeks | 4weeks | 8weeks | 12weeks |
|  | 10.01 | 7.81 | 7.85 | 8.13 |
|  | 10.05 | 7.58 | 7.81 | 8.19 |
|  | 10.11 | 7.19 | 7.21 | 7.89 |
|  | 11.02 | 8.05 | 8.1 | 8.53 |
|  | 11.16 | 8.15 | 8.29 | 8.71 |
