# Supplementary material for: Experimental study of tendon sheath repair via decellularized amnion to prevent tendon adhesion
Source: PLoS One. 2018 Oct 16;13(10):e0205811. doi: 10.1371/journal.pone.0205811 (PMC6191119; doi:10.1371/journal.pone.0205811)
Supplement: S2 Table — The original data of the total flexion angle of the toes were measured at 2, 4, 8, and 12 weeks after surgery. (DOCX) [file pone.0205811.s002.docx]

S2 Table: The total flexion angle of the toes. The original data of the total flexion angle of the toes were measured at 2, 4, 8, and 12 weeks after surgery.

| The total flexion angle of the toes（°） | | | | |
| --- | --- | --- | --- | --- |
| amniotic membrane group | 2weeks | 4weeks | 8weeks | 12weeks |
|  | 292.93 | 318.25 | 322.75 | 327.82 |
|  | 295.24 | 328.23 | 330.85 | 331.84 |
|  | 302.93 | 320.58 | 336.67 | 336.56 |
|  | 301.52 | 315.67 | 316.29 | 331.82 |
|  | 308.56 | 324.17 | 325.14 | 325.78 |
|  | | | | |
| medical membrane group | 2weeks | 4weeks | 8weeks | 12weeks |
|  | 302.34 | 314.26 | 306.25 | 303.63 |
|  | 290.17 | 322.56 | 304.74 | 308.31 |
|  | 292.37 | 311.71 | 318.94 | 317.62 |
|  | 307.38 | 330.65 | 319.47 | 321.64 |
|  | 309.63 | 327.24 | 322.63 | 325.42 |
|  | | | | |
| control group | 2weeks | 4weeks | 8weeks | 12weeks |
|  | 228.32 | 196.15 | 200.27 | 198.42 |
|  | 236.46 | 195.63 | 204.15 | 200.27 |
|  | 238.35 | 207.28 | 214.55 | 208.56 |
|  | 246.42 | 215.53 | 215.79 | 214.65 |
|  | 252.23 | 197.57 | 222.85 | 216.71 |
