# Supplementary material for: Experimental study of tendon sheath repair via decellularized amnion to prevent tendon adhesion
Source: PLoS One. 2018 Oct 16;13(10):e0205811. doi: 10.1371/journal.pone.0205811 (PMC6191119; doi:10.1371/journal.pone.0205811)
Supplement: S3 Table — The original data of The tendon maximum tensile breaking strength were measured at 2, 4, 8, and 12 weeks after surgery. (DOCX) [file pone.0205811.s003.docx]

S3 Table: The tendon maximum tensile breaking strength. The original data of The tendon maximum tensile breaking strength were measured at 2, 4, 8, and 12 weeks after surgery.

| The tendon maximum tensile breaking strength（N） | | | | |
| --- | --- | --- | --- | --- |
| amniotic membrane group | 2weeks | 4weeks | 8weeks | 12weeks |
|  | 13.54 | 52.34 | 106.45 | 152.52 |
|  | 18.87 | 51.85 | 97.41 | 150.25 |
|  | 18.45 | 53.28 | 105.58 | 149.54 |
|  | 15.41 | 56.79 | 104.14 | 155.47 |
|  | 14.79 | 56.94 | 98.26 | 153.63 |

|  | | | | |
| --- | --- | --- | --- | --- |
| medical membrane group | 2weeks | 4weeks | 8weeks | 12weeks |
|  | 12.16 | 42.26 | 85.12 | 145.31 |
|  | 13.48 | 41.35 | 84.11 | 145.24 |
|  | 14.26 | 44.42 | 86.87 | 146.24 |
|  | 19.61 | 49.57 | 89.85 | 151.39 |
|  | 19.41 | 50.61 | 89.81 | 152.54 |

|  | | | | |
| --- | --- | --- | --- | --- |
| control group | 2weeks | 4weeks | 8weeks | 12weeks |
|  | 12.26 | 30.24 | 50.24 | 146.89 |
|  | 13.16 | 32.24 | 52.06 | 148.22 |
|  | 15.32 | 35.83 | 56.64 | 149.68 |
|  | 20.32 | 36.69 | 57.93 | 151.75 |
|  | 21.59 | 37.87 | 58.85 | 152.91 |
